# Ct, *IL-18* polymorphism, and laboratory biomarkers for predicting chemosensory dysfunctions and mortality in COVID-19

**DOI:** 10.2144/fsoa-2022-0082

**Published:** 2023-03-09

**Authors:** Shukur Wasman Smail, Esmaeil Babaei, Kawa Amin

**Affiliations:** 1 Department of Biology, College of Science, Salahaddin University-Erbil, Iraq; 2 Department of Biology, School of Natural Sciences, University of Tabriz, Tabriz, Iran; 3 Department of Pharmacognosy, College of Pharmacy, Hawler Medical University, Erbil, Kurdistan Region, Iraq; 4 College of Medicine, University of Sulaimani, Sulaymaniyah, Iraq

**Keywords:** chemosensory dysfunctions, COVID-19, cyclic threshold, polymorphism and predictors

## Abstract

**Aim:**

Patients with COVID-19 often experience chemosensory dysfunction. This research intends to uncover the association of RT-PCR Ct value with chemosensory dysfunctions and SpO_2_. This study also aims to investigate Ct, SpO_2_, CRP, D-dimer, and -607 *IL-18* T/G polymorphism in order to find out predictors of chemosensory dysfunctions and mortality.

**Materials & methods:**

This study included 120 COVID-19 patients, of which 54 were mild, 40 were severe and 26 were critical. CRP, D-dimer, RT-PCR, and *IL-18* polymorphism were evaluated. **Results & conclusion**: Low Ct was associated with SpO_2_ dropping and chemosensory dysfunctions. *IL-18* T/G polymorphism did not show an association with COVID-19 mortality; conversely, age, BMI, D-dimer and Ct values did.

## Plain language summary

This research intends to uncover the association of RT-PCR Ct value with GD, OD, and SpO_2_. It also aims to investigate Ct, SpO_2_, CRP, D-dimer and -607 *IL-18* T/G polymorphism as predictors of chemosensory dysfunctions and mortality. This study included 120 COVID-19 patients, of which 54 were mild, 40 were severe and 26 were critical. Low Ct was associated with SpO_2_ dropping, GD, and OD. *IL-18* T/G polymorphism did not show an association with COVID-19 mortality, conversely, age, BMI, D-dimer and Ct values did.

## Graphical abstract

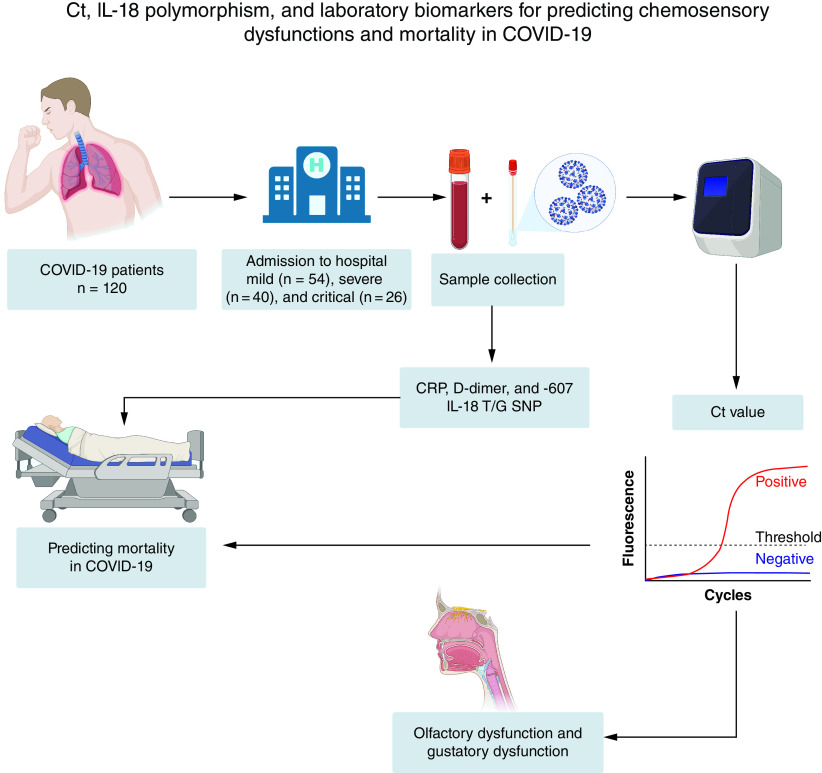

Wuhan, China was the origin of the SARS-CoV-2 which resulted in the COVID-19 [1]. The disease might be asymptomatic or produce mild, severe or critical symptoms [2]. Symptoms vary from individual to individual and country to country. Cough, myalgia, fever, and sore throat are the most prevalent symptoms [3]. The SARS-CoV-2 virus could attack the taste buds and olfactory epithelium, causing olfactory dysfunction (OD) and gustatory dysfunction (GD) [4]. GD comprises hypogeusia and dysgeusia, whereas OD comprises hyposmia and anosmia; their prevalence ranged from 5 to 88% [5–7]. In Spain, only 43.3% of COVID-19 have anosmia [8]. European populations have a higher incidence of OD compared with Asian populations [6,9]. Varying demographics may display different frequencies of OD because of discrepancies in the amount of ACE2 expressed in the olfactory epithelium [10,11]. The incidence of GD also varies because of the different expressions of ACE2 in the oral epithelium [12]. Variations in alcohol consumption and smoking habits between countries may result in a difference in ACE2 expression, which could lead to diverse signs and symptoms, such as chemosensory dysfunctions [13,14]. The single nucleotide polymorphism (SNP) of brain-derived neurotrophic factor (BDNF) is implicated in the degree of OD [15]. Polymorphisms in the *carbonic anhydrase VI* gene may be associated with the area-specific properties of early GD in COVID-19 patients and the taste-related effects of those who have recovered from COVID-19 [16].

The clinical laboratory has been a fundamental part of stratifying the severity and prognosis of the disease [17–19]. Systolic blood pressure and oxygen saturation (SpO_2_) were assessed alongside other biological measurements. It has been reported that the levels of D-dimer and C-reactive protein (CRP) are notably increased in individuals suffering from COVID-19 [20]. The gold standard technology of real-time polymerase chain reaction (RT-PCR) was utilized throughout the pandemic to diagnose SARS-CoV-2. Despite this, some scientists remain doubtful of this technology [21]. Moreover, cycle threshold (Ct) value in RT-PCR is also essential for interpretation. It is necessary to associate Ct value with disease severity to eliminate this doubt.

RT-PCR reaction's Ct value is the number of cycles at which the fluorescence of the PCR product is detectable above the background signal [22]. The amount of Ct cycles of RT-PCR must be calculated to inspect the virus in the sample. As a result, the Ct is an indirect measurement of the amount of RNA in the sample, meaning that a low Ct is associated with a high viral load and *vice versa* [23]. Unfortunately, the Ct value is not included in the lab report for the patient. The Ct value is a significant factor in the prognosis of COVID-19, however it is not included in the patient's laboratory report [23–25].

Interleukin-18 (IL-18), a pro-inflammatory cytokine, was present at a high level among COVID-19 patients. The positioning of the *IL-18* gene is on chromosome 11 in the q22.2–22.3 [26]. It is assumed that two SNPs can influence the expression of the *IL-18* gene and are linked to IL-18 concentration. Research has demonstrated that the SNPs located in the *IL-18* promoter region, such as rs187238 (-137 G >C) and rs1946518 (-607 C >A or T >G), can affect the expression of IL-18 [27]. Alteration of the -607 *IL-18* SNP influences the concentration of IL-18 in the body by modulating the cAMP response element-binding protein (CREB). There is evidence that the severity of COVID-19 is linked to high levels of IL-18 in the blood. The genetic variations of this cytokine may affect its expression and the amount present in the blood [28]. There are many papers explaining role of IL-6 and its polymorphism in prediction severity and mortality of COVID-19 [29–31], but there are few papers regarding role of *IL-18* SNP in prediction mortality of the disease.

The purpose of the research was to identify the relationship between Ct value with GD, OD, and SpO_2_ in patients with SARS-CoV-2 infection in Northern Iraq. We aimed to assess the importance of determining Ct value and SpO_2_ measurements upon admission in relation to regular laboratory markers (CRP and D-dimer), in order to predict COVID-19 mortality. According to our knowledge, no article has examined the association between the -607 T/G SNP of the *IL-18* gene and mortality in COVID-19. Therefore, this study was conducted to assess the association between the -607 T/G polymorphism of the *IL-18* gene and the mortality risk in the Kurdish population.

## Materials & methods

### Patients

From 1 May to 30 May 2021, 120 COVID-19 patients were recruited, with 60 (50%) males and 60 (50%) females, as displayed in Table 1. Participants of this study originated from several cities in Northern Iraq and displayed signs and symptoms corresponding to SARS-CoV-2 infection. The infection was confirmed via RT-PCR. As per Chinese guidelines, the COVID-19 participants were categorized into three groups: “mild” 54 (45%), “severe” 40 (33.33%), and “critical” 26 (21.67%) [32,33]. The protocol that divided COVID-19 patients into mild, moderate, severe, and critical groups was slightly modified for the current study; the mild and moderate groups were merged into the mild group. According to this protocol, mild cases either don't exhibit pneumonia or exhibit mild pneumonia; severe cases manifest pneumonia characterized by shortness of breath and their SpO_2_ <93 and critical patients suffer from respiratory failure, septic shock, or multiple organ failure [33].

**Table 1.** Comparison of demographic characteristics and serum markers among groups of COVID-19 patients.

Parameters	Mild (mean ± SEM)	Severe (mean ± SEM)	Critical (mean ± SEM)	p-value
n	54	40	26	–
Gender (M/F)	30/24	22/18	8/18	–
Age (Years)	39.44 ± 2.191	48.10 ± 2.570	58.54 ± 2.839	<0.0001
BMI (kg/m^2^)	26.79 ± 0.781	30.54 ± 1.326	29.96 ± 1.573	0.020
D-dimer (μg/ml)	0.450 ± 0.100	10.12 ± 3.284	46.60 ± 7.883	<0.0001
SpO_2_ (%)	94.15 ± 0.901	63.85 ± 4.311	49.08 ± 3.847	<0.0001
CRP (mg/l)	5.200 ± 2.672	67.20 ± 13.07	65.82 + 8.615	<0.0001
Ct value	24.65 ± 0.6444	23.25 ± 0.593	15.64 ± 2.139	<0.0001

Comparison among groups was done via one-way ANOVA. Tukey test was applied as a post-hoc test for multiple comparisons. Data were presented as mean ± standard error of the mean (SEM), Probability (p)-value less than 0.05 was considered significant.

CRP: C-reactive protein; Ct: Cyclic threshold; F: Female; M: Male; SpO_2_: Oxygen saturation level.

During the thirty-day follow-up, all patients in the critical group passed away in the hospital. All COVID-19 patients were non-vaccinated; the patients did not involve in any therapeutic intervention. For the purpose of genotyping, the COVID-19 study participants were re-categorized into two groups based on mortality: those who survived (mild and severe) (n = 94) and those who did not survive (critical) (n = 26). Patients who did not have access to clinical information or did not have a blood or swab sample were excluded.

### Sample & data collection

Blood samples were taken from all the participants prior to the administration of any drugs. An oro-nasopharyngeal swab (eSwab, Copan, USA) was taken from the back of the throat and the nasopharynx to investigate the SARS-CoV-2 virus [34]. The emergency departments of the hospital provided data concerning COVID-19, such as comorbidities, demographics, and medical history. In terms of comorbidities, COVID-19 patients were found to have hypertension (HTN), diabetes mellitus (DM), rheumatoid arthritis (RA), and cancer. 78 patients (65%) didn't have any other underlying disease. While 42 patients (35%) had a personal history of comorbidity. HTN was found to be the most regularly occurring comorbidity among COVID-19 cases, with 42.85% (n = 18). DM, on the other hand, was seen in 19.08% of cases (n = 8). Ten people with COVID-19 (23.81%) reported having more than two comorbidities. Cancer was seen in two cases, with a rate of 4.76 percent, while other diseases occurred in four cases at a rate of 9.53 percent.

### Methods for measurement of parameters

D-dimer and CRP were evaluated by Cobas c311 (Roche, Germany). A Masimo pulse oximeter was utilized to measure SpO_2_. The SARS-CoV-2 detection was conducted via RT-PCR (Bio-Rad, USA); all the procedures from RNA extraction, cDNA synthesis, and virus detection in the swab were done by Bio-Rad reliance SARS-CoV-2 RT-PCR assay kit (Bio-Rad, USA).

### DNA extraction & genotyping of *IL-18* rs1946518 SNP

As instructed by the manufacturer, a DNA extract kit (Qiagen, Germany) was used to extract DNA from whole blood. The primers used in this study were designed on this website: http://primer1.soton.ac.uk/primer1.html.

Outer forward: 5“CCTACAATGTTACAACACTTAAAAT”3

Outer reverse: 5“ATAAGCCCTAAATATATGTATCCTTA”3

Inner forward: 5“GATACCATCATTAGAATTTTGTG”3

Inner reverse: 5“GCAGAAAGTGTAAAAATTATCAA”3

The 20 μL reaction tube for the PCR procedure included 2 μL of genomic DNA, 10 μL of mastermix (Ampliqon, Denmark), 1 μL of each primer, and 4 μL of deionized distilled water. The thermocycling protocol began with an initial denaturation step at 94°C for 7 minutes, followed by 30 cycles of 94°C for 40 seconds, 54°C for 40 seconds, 72°C for 1 minute, and a final extension of 72°C for 5 minutes.

The PCR products were then visualized on a 2% agarose gel electrophoresis stained with ethidium bromide and examined under ultraviolet light (Brown, 2016). Gel electrophoresis results of the TG genotype revealed three bands (440 bp, 208 bp, 278 bp), the TT genotype yielded two bands (440 bp and 208 bp) and the GG genotype displayed two bands (440 bp and 278 bp) (Supplementary Figure 1). For ensuring of the result, 20% of samples were sent for sanger sequencing (Macrogen, Korea), only outer forward and reverse primers were used for sequencing. The result of the sanger sequence was shown on the NCBI via the accession code (OP896193 and OP896194) (not released yet). It was analyzed via the Geneious prime program and confirmed by NCBI nucleotide blast (Supplementary Figure 2). NC_000011.10 was used as a reference sequence (https://www.ncbi.nlm.nih.gov/nucleotide/NC_000011.10?report=genbank&log$=nuclalign&blast_rank=1&RID=SCAV7SFP01N).

### Statistical analysis

The SPSS 28 (IBM, USA), GraphPad Prism 9 (GraphPad Software, Inc., USA), and MedCalc 20 (MedCalc Software, Ltd., Belgium) were used for statistical analysis and making graphs. All of the data was found to be in accordance with the Shapiro-Wilk and D'Agostino normality tests, as well as the Levene's test which showed homogeneity of variance. A two-group comparison was conducted using an independent *t*-test, while a one-way ANOVA was used for comparing more than two groups. A Tukey test was implemented as a post-hoc test for multiple comparisons.

Pearson correlation analysis was used to determine linear relationships between quantitative variables. Binary univariate logistic and multiple regression were used for the prediction of variables in severity and mortality. The receiver operating characteristic (ROC) curve was utilized in order to predict mortality. The mortality prognosis of laboratory parameters was established by means of the cut-off (with the use of Youden index) from the ROC curve. The predictive accuracy of biomarkers was assessed through positive predictive value (PPV) and negative predictive value (NPV). The Chi-square (χ^2^) test was applied to assess the COVID-19 genotype and allele frequencies. A p-value of 0.05 or below was considered being statistically significant.

## Results

### Comparison of demographics & laboratory markers

**Figure 1.**
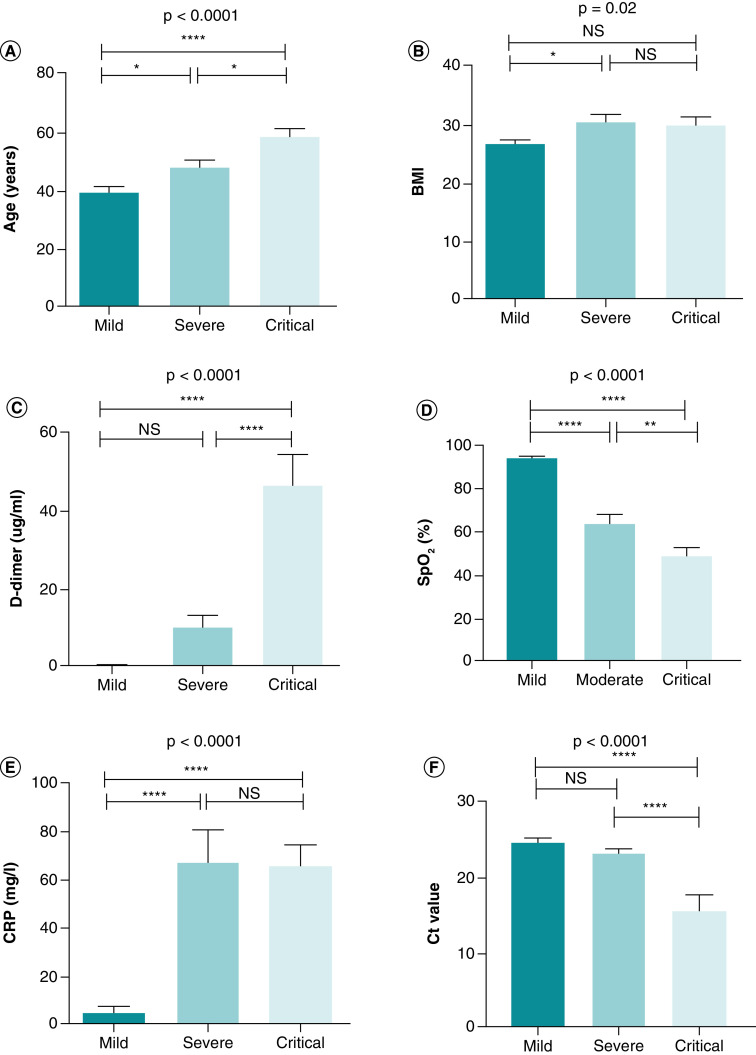
Comparison of variables among groups of COVID-19 patients. One-way ANOVA was used for the comparison of **(A)** Age. **(B)** BMI. **(C)** D-dimer. **(D)** SpO_2_. **(E)** CRP. **(F)** Ct value. Tukey test was applied as a post-hoc test for multiple comparisons. *p-value < 0.05; **p-value < 0.01; ***p-value < 0.001 and ****p-value < 0.0001. CRP: C-reactive protein; Ct: Cyclic threshold; D-dimer: Degradation product of fibrin; NS: Non-significant; SpO2: Oxygen saturation.

Age and CRP were much higher in the severe and critical groups of COVID-19 than in the mild group (Table 1, Figure 1a and e). Concerning BMI, our results showed that statistical significance (p = 0.02) was only found between the severe and mild groups (Table 1, Figure 1b). In the mild, severe, and critical groups, the average amount of D-dimer was (0.450 ± 0.100), (10.12 ± 3.284), and (46.60 ± 7.883), respectively. The difference in D-dimer between the mild and severe groups was not statistically significant, but there were significant differences between the other categories (Table 1, Figure 1c). Compared with the mild group, severe and critical COVID-19 showed a significant (p < 0.0001) drop in SpO_2_ (Table 1, Figure 1d).

### Comparison of Ct value among groups of COVID-19 patients

**Figure 2.**
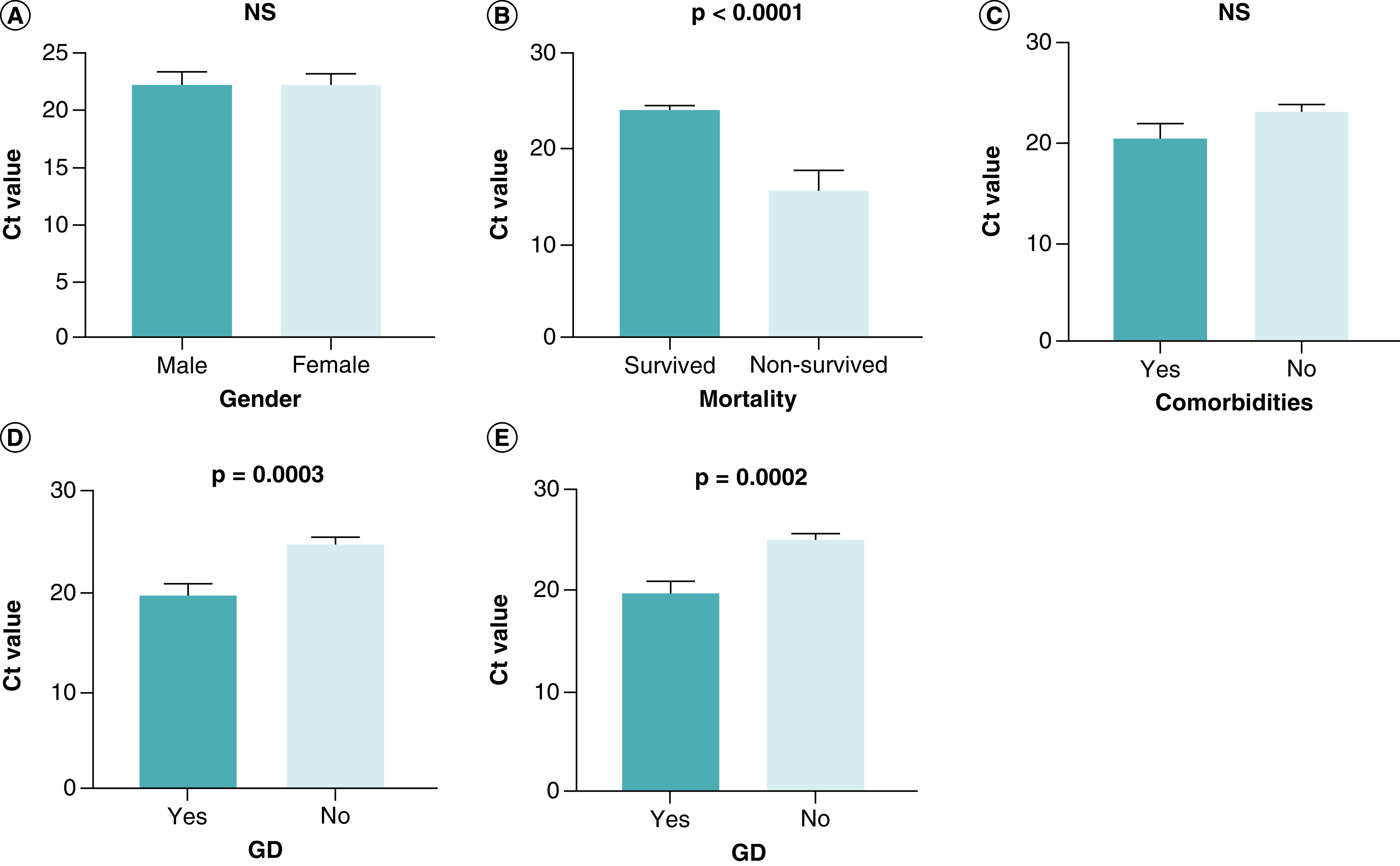
Comparison of Ct value between groups. An independent *t*-test was used for the comparison of Ct value between groups of **(A)** gender (male vs female), **(B)** mortality (survived vs non-survived), **(C)** comorbidities (yes vs no), **(D)** GD (yes vs no), and **(E)** OD (yes vs no). A probability (p)-value of less than 0.05 was considered significant.Ct: Cyclic threshold; GD: Gustatory dysfunction; OD: Olfactory dysfunction; NS: Non-significant.

Among COVID-19 patients, the Ct value decreased significantly in the critical groups compared with the mild and severe groups (p = 0.025 and p < 0.0001, respectively). In contrast, there were no statistically significant differences in the Ct value between the mild and severe groups (p = 0.541) (Table 1, Figure 1). Ct values for male patients (22.22 ± 1.113) and female patients (22.24 ± 0.961) were not significantly different (p = 0.992) (Table 2, Figure 2a). Patients who survived had a Ct value of 24.05 0.455, while those who did not had a Ct value of 15.64 ± 2.139. There was a statistically significant difference (p < 0.0001) between them (Table 2, Figure 2b). Comorbid patients had a mean Ct value of 20.54 ± 1.461, while non-comorbid patients had a mean Ct value of 23.14 ± 0.776; there was no significant difference between them (p = 0.089) (Table 2, Figure 2c).

**Table 2.** Clinical characteristics and symptoms of COVID-19 patients and Ct value of RT-PCR.

Variables	n (%)	Ct value (mean + SEM)	p-value
Gender			
Male	60 (50%)	22.22 ± 1.113	0.992
Female	60 (50%)	22.24 ± 0.961	
Status			
Survived	94 (78.33%)	24.05 ± 0.455	<0.0001
Non-survived	26 (21.67%)	15.64 ± 2.139	
Comorbidities			
Yes	42 (35%)	20.54 ± 1.461	0.089
No	78 (65%)	23.14 ± 0.776	
GD			
Yes	64 (53.33%)	19.84 ± 1.092	0.0003
No	56 (46.67%)	24.96 ± 0.639	
OD			
Yes	64 (53.33%)	19.80 ± 1.099	0.0002
No	56 (46.67%)	25.01 ± 0.606	

Comparison between groups was done via an independent *t*-test. Data were presented as mean ± SEM, p-value less than 0.05 was considered significant.

Ct: Cyclic threshold; GD: Gustatory dysfunction; n: Number of participants; OD: Olfactory dysfunction; SEM: Standard error of the mean.

The mean Ct value of COVID-19 patients with GD was 19.84 ± 1.092 and that of patients without GD was 24.96 ± 0.639; there was a significant difference between them (p = 0.0003) (Table 2, Figure 2d). Patients with OD had a mean Ct value of 19.80 ± 1.099, while those without OD had a mean Ct value of 25.01 ± 0.606. A substantial difference was observed between their Ct values (p = 0.0002) (Table 2, Figure 2e).

### Binary univariate regression model for GD & OD

Ct value was incorporated into a binary univariate logistic regression model to predict OD and GD. RT-PCR Ct values are independent predictors of OD and GD. As shown in Table 3, our model revealed a significant relationship between the Ct value and GD (p = 0.001, OR = 0.700) and OD (p = 0.001, OR = 0.688). A one-unit increase in the Ct value reduces the probability of an individual having GD 1.43 (1/0.7) times, and the probability of OD to be 1.45 (1/0.68) times.

**Table 3.** Binary univariate Logistic regression to use Ct value as a predictor for GD and OD.

Dependent variables	Independent variables	B	OR	95% CI for OR	p-value
GD	Ct value	-0.357	0.700	0.566–0.865	0.001
OD	Ct value	-0.374	0.688	0.554–0.855	0.001

Binary univariate logistic regression was used for utilizing the Ct value as a predictor for GD or OD in COVID-19 patients. Probability (p)-value less than 0.05 was considered significant.

B: Regression coefficient; CI: Confidence interval; Ct: Cyclic threshold; GD: Gustatory dysfunction; OD: Olfactory dysfunction; OR: Odds ratio, SEM: Standard error of the mean.

### Multiple linear regression model for SpO_2_

To find out if there is an association between lab parameters and the risk of SpO_2_ dropping. The models were made with multiple linear regression, and the “stepwise” method was used to choose the variables that went into the models and acted as predictors. The calibration of the model was checked by determining whether the related variables had multicollinearity (tolerance and variance inflation factor (VIF)). By doing stepwise multiple linear regression analysis on age, BMI, D-dimer, CRP, Ct value, and SpO_2_, we found that model 1 (Ct value alone), model 2 (Ct value and age), model 3 (Ct value, age, and BMI), and model 4 (Ct value, age, BMI, and D-dimer) were strongly associated to SpO_2_ dropping in COVID-19 patients. Simultaneously, the inclusion of CRP did not enhance the regression model, and as a result, it was omitted from the stepwise analysis. As shown in Table 4, age, BMI, and D-dimer were predictors for SpO_2_ dropping. While the Ct value could predict the rise in SpO_2_.

**Table 4.** Multiple linear regression of Ct value, age, BMI, D-dimer and CRP to predict SpO_2_ level in COVID-19 patients.

Dependent variable	Models	Independent variables	B	p-value
SpO_2_	Model 1	Ct value	2.435	<0.0001
	Model 2	Ct value	1.893	<0.0001
		Age	-0.546	0.005
	Model 3	Ct value	1.674	<0.0001
		Age	-0.509	0.007
		BMI	-1.039	0.018
	Model 4	Ct value	1.215	0.013
		Age	-0.422	0.023
		BMI	-1.238	0.005
		D-dimer	-0.233	0.037

Multiple linear regression was done by SPSS 27. In stepwise linear regression, model 1 (Ct) and model 2 (Ct value + age), model 3 (Ct value + age + BMI) and model 4 (Ct value + age + BMI + D-dimer) were considered predictors for SpO_2_ depression in COVID-19. CRP did not enhance the regression model, and as a result, it was excluded from the stepwise analysis. Probability (p)-value less than 0.05 was considered significant.

B: Regression coefficient; Ct: Cyclic threshold; D-dimer: Degradation product of fibrin; SpO_2_: Oxygen saturation level.

### The correlation between the variables

**Figure 3.**
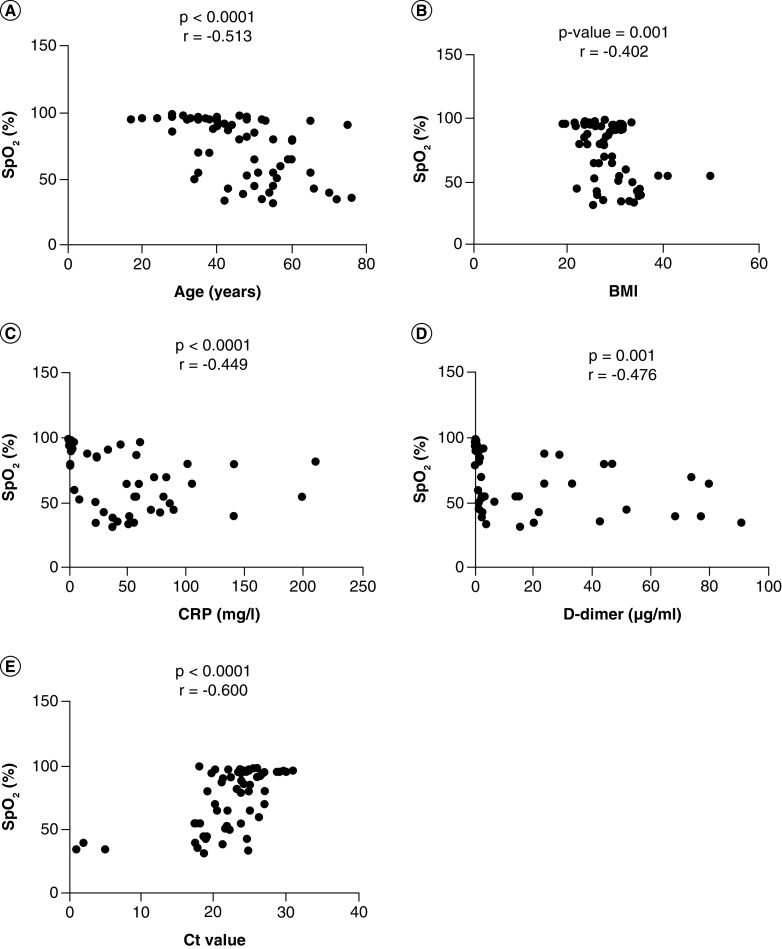
Correlation of SpO_2_ with other variables. A Pearson correlation test was used for the association of SpO_2_ with **(A)** Age, **(B)** BMI, **(C)** CRP, **(D)**, D-dimer, **(E)** Ct value. A probability (p)-value less than 0.05 was considered significant, correlation coefficient (r) shows the degree of correlation. CRP: C-reactive protein; Ct: Cyclic threshold; D-dimer: Degradation product of fibrin; SpO_2_: Oxygen saturation.

The Pearson correlation was done between variables (age, D-dimer, CRP, BMI, Ct value, and SpO_2_). The significant correlation coefficients (r) were found between SpO_2_ and Ct value (r = -0.600, p < 0.0001), SpO_2_ and age (r = -0.513, p < 0.0001), CRP and SpO_2_ (r = -0.449, p < 0.0001), SpO_2_ and D-dimer (r = -0.476, p = 0.001) and SpO_2_ and BMI (r = -0.402, p = 0.001). The detailed correlations can be seen in Table 5, Figure 3.

**Table 5.** Correlation among various variables (Ct value, age, BMI, D-dimer, CRP, and SpO_2_).

Variables		SpO_2_	Ct	Age	CRP	D-dimer	BMI
r	SpO_2_	1.000	0.600	-0.513	-0.449	-0.476	-0.402
	Ct	0.600	1.000	-0.423	-0.306	-0.511	-0.260
	Age	-0.513	-0.423	1.000	0.292	0.376	0.182
	CRP	-0.449	-0.306	0.292	1.000	0.408	0.335
	D-dimer	-0.476	-0.511	0.376	0.408	1.000	-0.031
	BMI	-0.402	-0.260	0.182	0.335	-0.031	1.000
p-value (1-tailed)	SpO_2_	–	0.000	0.000	0.000	0.000	0.001
	Ct	0.000	–	0.000	0.009	0.000	0.022
	Age	0.000	0.000	–	0.012	0.002	0.082
	CRP	0.000	0.009	0.012	–	0.001	0.004
	D-dimer	0.000	0.000	0.002	0.001	–	0.408
	BMI	0.001	0.022	0.082	0.004	0.408	–

A Pearson correlation was done by SPSS 27. Probability (p)-value less than 0.05 was considered significant.

BMI: Body mass index; CRP: C-reactive protein; Ct: Cyclic threshold; D-dimer: A degradation product of fibrin; r: Pearson correlation coefficient, SpO_2_: Oxygen saturation.

### ROC curve analysis as a predictor of mortality in COVID-19

**Figure 4.**
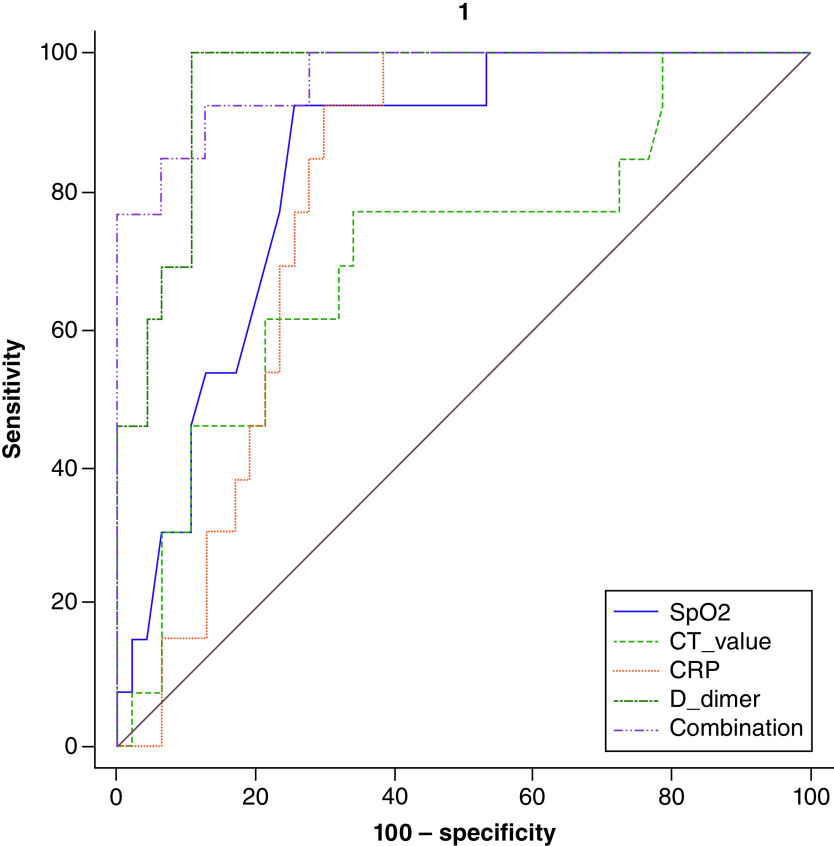
ROC curve analysis of Ct value, D-dimer, CRP, SpO_2_ and their combinations to predict mortality of SARS-CoV-2 infection. The ROC curve figure was created by MedCalc 20. The COVID-19 patients were divided into survived and non-survived considering status in the ROC curve analysis. CRP: C-reactive protein; Ct: Cyclic threshold; D-dimer: Degradation product of fibrin; SpO_2_: Oxygen saturation; ROC: Receiver operating characteristic curve.

In Table 6 and Figure 4, ROC curves were constructed for the predicting mortality of 120 COVID-19 patients. Areas under the curve (AUC) have been reported for the predicting mortality of COVID-19, which is equal to 0.899, 0.956, 0.797, 0.879, and 0.989, for Ct value, D-dimer, CRP, SpO_2_, and their combinations, respectively. The results also showed that the AUC with the best performance in biomarkers was the combination of Ct value, D-dimer, CRP, and SpO_2_. They allow us to predict the mortality of the disease with a sensitivity of 92.31% and a specificity of 100%; their NPV and PPV were 97.9 and 100%, respectively. A Ct value of 20.47 is the cutoff point for hospital mortality prediction. The ideal cutoff point for predicting mortality for D-dimer, CRP, and SpO_2_ was 6.81μg/ml, 33.56 mg/l, and 70%, respectively.

**Table 6.** ROC curve analysis of Ct value, D-dimer, CRP, SpO_2_, and their combinations to predict mortality of SARS-CoV-2 infection.

	Cut-off	AUC	95% CI	p-value	Sensitivity	95% CI	Specificity	95% CI	PPV	95% CI	NPV	95% CI
Ct value	≤20.47	0.899	0.793–0.961	<0.0001	84.62	54.6–98.1	85.11	71.7–93.8	61.1	43.3–76.4	95.2	84.8–98.6
D-dimer (μg/ml)	>6.81	0.956	0.869–0.992	<0.0001	100.00	75.3–100.0	89.36	76.9–96.5	72.2	53.2–85.6	100.0	–
CRP (mg/l)	>33.56	0.797	0.673–0.890	<0.0001	92.31	64.0–99.8	70.21	55.1–82.7	46.2	35.0–57.7	97.1	83.3–99.5
SpO_2_ (%)	≤70	0.879	0.769–0.949	<0.0001	100.00	75.3–100.0	74.47	59.7–86.1	52.0	39.9–63.8	100.0	–
Combinations	>0.371	0.989	0.919–1.000	<0.0001	92.31	64.0–99.8	100.00	92.5–100.0	100.0	–	97.9	87.7–99.7

ROC curve analysis was done by MedCalc 20. The COVID-19 patients were divided into survived and non-survived considering as status in the ROC curve analysis.

AUC: Area under curve; CI: Confidence interval; CRP: C-reactive protein; Ct: Cyclic threshold; D-dimer: Degradation product of fibrin; SpO_2_: Oxygen saturation; NPV: Negative predictive value; PPV: Positive predictive value; ROC: Receiver operating characteristic curve.

Probability (p)-value less than 0.05 was considered significant.

### Association of - 607 *IL-18* SNP to susceptibility to SARS-CoV-2 infection

In this study, we analyzed the *IL-18* polymorphism between survivors and non-survivors of COVID-19. 61.54% of non-survivors (16 out of 26) and 67.02% of survivors from COVID-19 (63 out of 94), can be identified as heterozygous (TG) for the rs1946518 *IL-18* polymorphism. 26.92% of non-survivors and 27.66% of survivors from COVID-19 were homozygous for the TT genotype. Regarding the GG genotype, 11.54% of the non-survivors were homozygous for it, and 5.32% of the COVID-19 survivors showed polymorphism in homozygosity. When performing the χ^2^, we found that the *IL-18* TG and GG genotypes were not significantly associated with increased mortality of COVID-19 compared with the *IL-18* TT genotypes (p = 0.9, OR = 1.060, 95% CI = 0.413–2.764) and (p = 0.378, OR = 0.449, 95% CI = 0.094–2.063) respectively. There was no significant association in the comparison of the dominant model (TT + TG vs GG: OR = 0.431, 95% CI: 0.102–1.736; p = 0.368) and the recessive model (TT vs TG/GG: OR = 0.964, 95% CI: 0.379–2.478; p = 0.9) (Table 7).

**Table 7.** The genotypes and allele distribution of -607 T >G (rs1946518) polymorphism in non-survivor and survivor COVID-19.

Polymorphism	Non-survivor (n = 26)		Survivor (n = 94)		OR	95% CI	p-value
	n	%	n	%			
TT	7	26.92	26	27.66	1	–	–
TG	16	61.54	63	67.02	1.060	0.413–2.764	0.9
GG	3	11.54	5	5.32	0.449	0.094–2.063	0.378
TG + GG	19	73.08	68	72.34	0.964	0.379–2.478	0.9
TT + TG	23	88.46	89	94.68	0.431	0.102–1.736	0.368
T	30	57.69	115	61.17	0.866	0.476–1.635	0.749
G	22	42.31	73	38.83			

Chi-square (χ^2^) was used for associating genotypes and alleles between groups.

A probability (p)-value of less than 0.05 was considered significant.

CI: Confidence interval; OR: Odds ratio.

Furthermore, the carrier frequency of the T allele was not significantly lower in the non-survivor group (57.69%) compared with the survivor COVID-19 group (61.17). While the carrier frequency of the G allele was not significantly higher in the non-survivor (42.31%) compared with the survivor's COVID-19 (38.83%). Finally, there was no statistical difference in the frequencies of the two alleles (T vs G: OR = 0.866, 95% CI: 0.476–1.635; p = 0.749) (Table 7).

## Discussion

The respiratory and cardiovascular systems are the primary target of SARS-CoV-2, yet, there is a significant amount of proof to support the importance of chemosensory dysfunctions when assessing and diagnosing COVID-19 patients. The decreased sense of taste and smell in the current COVID-19 pandemic strongly shows a SARS-CoV-2 infection. Furthermore, in addition to standard laboratory values (D-dimer and CRP), parameters such as SpO_2_ and the Ct value of the RT-PCR should be considered [35–38].

Our results revealed the patients who exhibited severe and critical symptoms had a higher CRP than mild cases of COVID-19, CRP levels were associated negatively with SpO_2_; a published paper confirms that this result reported that an exaggerated elevation of CRP in patients with COPD was negatively correlated with SpO_2_ [39]. Despite elevation of CRP in critical COVID-19 patients, but our regression model didn't support its use as predictor for mortality, this finding is in contrast to Huyut MT, Ilkbahar F [40] and Xie J*et al.* [41] who displayed that CRP was an important predictor for the progression of COVID-19 disease.

D-dimer levels increased in COVID-19's severe and critical groups. This result parallels a published paper by Yu H-H*et al.* [42], who documented that D-dimer was higher in patients suffering from severe COVID-19 [42]. D-dimer levels correlated negatively with SpO_2_; therefore, D-dimer may be a precise target to be assessed to indicate the mortality, as seen in the ROC curve analysis of this study. Yalçin, 2020 also confirmed our result; D-dimer and SpO_2_ were shown to be negatively correlated by him [43]. Huyut MT*et al.* [44] also documented that D-dimer was a predictor for mortality in COVID-19.

A low Ct value was found in COVID-19 patients suffering from GD and GD. This result is parallel with many papers that were done in this field [45–47]. There are many explanations for virus-induced GD and OD. First, the virus induces stomatitis and rhinitis, that leads to the breakdown of the neurosensory cells by the antibody. Second, the virus attacks the central nervous system, peripheral nervous system, cerebral cortex, and exclusively cranial nerves related to taste and smell. Lastly, the virus attacks directly ACE_2_ expressed on olfactory epithelium and taste buds [48]. The Ct value did not show the gender-based difference in the current study. This result coincides with another finding [49]. However, in COVID-19, The males have been infected more severely than females [50]; the increased mortality in a male might not be because of the viral load, but it may be other reasons: first, the bad habits of males such as smoking and drinking [51]; second, the testosterone in the male has immunosuppressive activity and increases the expression of TMPRSS_2_ which is the co-receptor for binding SRAS-CoV-2 virus. In addition, the estrogen in the female has immune-boosting properties against the virus [52]. There was a variation in the immune response dependent on gender. Suppressing toll-like receptor (TLR)-induced interferon (IFN) release decreases the male's capability to eradicate the virus [53,54].

The Ct value was lower in non-survivor patients in this study since the high load of the virus may lead to damage to the lung and induce pneumonia [55]. The multiple regression and correlation in this study showed that the Ct value associated with the dropping level of SpO_2_. There are many challenges in adding CT values to clinical reports: there is no industry-wide standard cut-off; it may vary depending on the type of commercial kits and the stage of the disease; and finally, the ability of the healthcare provider taking the swab and the COVID-19 patients' tolerance may also affect CT values [56]. After addressing these issues and enhancing RT-PCR’ sensitivity, adding the Ct value to the results of the test might be an excellent decision.

SpO_2_ assesses the respiratory function and arterial oxygenation of COVID-19 and its level decreased in severe and critical groups compared with mild groups. Correlation showed that age, BMI, CRP, and D-dimer were negatively associated, while Ct value was positively associated with a level of SpO_2_. The reason for the association of age with COVID-19 mortality could be due to the presence of comorbidities in older age [57]. An age related malfunction in T-lymphocyte and B-lymphocyte cells results in their inability to clear the virus [58]. Obesity is one of the risk factors for increased mortality in COVID-19 [59,60]. When COVID-19 patients have a high BMI, pro-inflammatory cytokines are also increased, causing damage to lung cells and a decline in SpO_2_ levels [61–63].

This study evaluated the association of polymorphisms of - 607 SNP of *IL-18* with COVID-19 mortality among the Kurdish population. The - 607 SNP of *IL-18* changes the immune response to many viruses, including hepatitis B virus [64], hepatitis C virus, cytomegalovirus [65], and human immune deficiency virus [66]. The cytokine genes are very polymorphic that may change the concentration of cytokine; *IL-18* has - 607 SNP in the promoter that causes elevation of IL-18. Serum elevation of IL-18 can be regarded as a bad prognostic factor in COVID-19 in the Brazilian population [67]. The current study documented that - 607 SNP of *IL-18* was not linked to mortality in COVID-19. Chen W-J*et al.* [68] proved that the TG genotype of - 607 SNP of *IL-18* was associated with viral shedding in SARS-CoV-1.

This study has some limitations. First, the sample size is relatively small, if the sample was taken from all governments in Iraq so that the sample represents the population of Iraq. Second, the blood was only taken at the time of admission; it was better to retake blood at regular intervals. Third, healthy controls were not enrolled in the current study. Finally, reports of the prevalence of olfactory and taste disturbances in patients with SARS-CoV-2 have been based mainly on questionnaires, which may overestimate the association of these alterations with this disease.

Future studies should measure SpO_2_, Ct value, GD, and OD changes in a follow-up study at early infection and monitor their levels during several stages of disease progression. Despite evidence of the existence of alteration of taste and smell as a symptom of COVID-19, it is still required to carry out experimental studies that explain the mechanism by which the infection produces this wide range of alterations, as well as their exact prevalence to find effective strategies for prevention, diagnosis, treatment, and rehabilitation of these conditions.

## Conclusion

The leading utility of our finding may assist physicians in focusing on measuring SpO_2_, Ct beside D-dimer at the early stage of the disease since both parameters were significantly decreased in COVID-19 patients with a poor prognosis at the time of hospital admission. Besides involving Ct in the mortality of COVID-19, a low value of Ct value is also associated with GD and OD. Age and BMI were also predictors of COVID-19 mortality. Therefore, individuals with low SpO_2_ and Ct values but high D-dimer should be monitored carefully to prevent death. The current study's findings suggest that Ct value and its interpretation comments on RT-PCR's report can be used as an early warning system to predict mortality in COVID-19. Once the challenges of Ct value have been overcome, Ct values can be utilized in conjunction with other laboratory biomarkers and clinical characteristics to manage the disease. -607 *IL-18* T/G SNP was not associated with mortality in COVID-19. It may be of great diagnostic and therapeutic importance to understand the genetic basis of COVID-19.

**Summary points**

- It is essential to document the Ct value in the clinical report at the time of admission, since a low Ct value is linked to a higher risk of mortality.
- Ct value is also linked to OD and GD.
-607 *IL-18* T/G polymorphism was not associated with mortality in COVID-19 patients.
- The regression model suggested that Ct value, age, BMI and D-dimer were predictors of a decrease in SpO_2_.

## References

[1] Lekhraj Rampal M, Seng LB. Coronavirus disease (COVID-19) pandemic. Med. J. Malays 75(2), 95 (2020). 32281587

[2] Marshall JC, Murthy S, Diaz J A minimal common outcome measure set for COVID-19 clinical research. Lancet Infect Dis 20(8), e192–e197 (2020). 32539990 10.1016/S1473-3099(20)30483-7 PMC7292605

[3] WHO. Coronavirus disease (COVID-19). https://www.who.int/health-topics/coronavirus#tab=tab_3 (March 24, 2020).

[4] Lechien JR, Chiesa-Estomba CM, De Siati DR Olfactory and gustatory dysfunctions as a clinical presentation of mild-to-moderate forms of the coronavirus disease (COVID-19): a multicenter European study. Eur Arch Oto-Rhino-L 277(8), 2251–2261 (2020). 10.1007/s00405-020-05965-1 PMC7134551 32253535

[5] Haehner A, Draf J, Dräger S, Hummel T. Predictive value of sudden olfactory loss in the diagnosis of COVID-19. ORL 82(4), 175–180 (2020). 32526759 10.1159/000509143 PMC7360503

[6] Menni C, Valdes AM, Freidin MB Real-time tracking of self-reported symptoms to predict potential COVID-19. Nat. Med. 26(7), 1037–1040 (2020). 32393804 10.1038/s41591-020-0916-2 PMC7751267

[7] Mao L, Jin H, Wang M Neurologic manifestations of hospitalized patients with coronavirus disease 2019 in Wuhan, China. JAMA Neurol 77(6), 683–690 (2020). 32275288 10.1001/jamaneurol.2020.1127 PMC7149362

[8] Pollán M, Pérez-Gómez B, Pastor-Barriuso R Prevalence of SARS-CoV-2 in Spain (ENE-COVID): a nationwide, population-based seroepidemiological study. Lancet 396(10250), 535–544 (2020). 32645347 10.1016/S0140-6736(20)31483-5 PMC7336131

[9] Chiesa-Estomba CM, Lechien JR, Saussez S. The alteration of smell and taste in COVID-19 patients. A diagnostic resource in primary care. Aten Primaria 52(8), 592–593 (2020). 32620292 10.1016/j.aprim.2020.05.005 PMC7250780

[10] Desforges M, Le Coupanec A, Dubeau P, Bourgouin A, Lajoie L, Dubé M. Coronavirus humanos y otros virus respiratorios:¿ patógenos oportunistas subestimados del sistema nervioso central? Virus 12(1), 14 (2019).

[11] Cao Y, Li L, Feng Z Comparative genetic analysis of the novel coronavirus (2019-nCoV/SARS-CoV-2) receptor ACE2 in different populations. Cell Discov 6(1), 1–4 (2020). 32133153 10.1038/s41421-020-0147-1 PMC7040011

[12] Xu H, Zhong L, Deng J High expression of ACE2 receptor of 2019-nCoV on the epithelial cells of oral mucosa. Int J Oral Sci 12(1), 1–5 (2020). 32094336 10.1038/s41368-020-0074-x PMC7039956

[13] Huyut M, Soygüder S. The multi-relationship structure between some symptoms and features seen during the new coronavirus 19 infection and the levels of anxiety and depression post-Covid. East J Med 27(1), 1–10 (2022).

[14] Friske MM, Giannone F, Senger M, Seitz R, Hansson AC, Spanagel R. Chronic alcohol intake regulates expression of SARS-CoV2 infection-relevant genes in an organ-specific manner. Alcohol. Clin. Exp. Res. (2023). 10.1111/acer.14981 36774629

[15] Roh D, Lee D-H, Kim SW The association between olfactory dysfunction and cardiovascular disease and its risk factors in middle-aged and older adults. Sci Rep 11(1), 1248 (2021). 33441955 10.1038/s41598-020-80943-5 PMC7806612

[16] Tsuchiya H. Gustatory and Saliva Secretory Dysfunctions in COVID-19 Patients with Zinc Deficiency. Life 12(3), 353 (2022). 35330104 10.3390/life12030353 PMC8950751

[17] Mertoglu C, Huyut MT, Arslan Y, Ceylan Y, Coban TA. How do routine laboratory tests change in coronavirus disease 2019? Scand J Clin Lab Invest 81(1), 24–33 (2021). 33342313 10.1080/00365513.2020.1855470

[18] Huyut MT, Üstündağ H. Prediction of diagnosis and prognosis of COVID-19 disease by blood gas parameters using decision trees machine learning model: a retrospective observational study. Medical Gas Res 12(2), 60 (2022). 10.4103/2045-9912.326002 PMC8562394 34677154

[19] Huyut MT, Huyut Z. Forecasting of Oxidant/Antioxidant levels of COVID-19 patients by using Expert models with biomarkers used in the Diagnosis/Prognosis of COVID-19. Int Immunopharmacol 100(5), 108127 (2021). 34536746 10.1016/j.intimp.2021.108127 PMC8426260

[20] Ali AM, Rostam HM, Fatah MH, Noori CM, Ali KM, Tawfeeq HM. Serum troponin, D-dimer, and CRP level in severe coronavirus (COVID-19) patients. Immun Inflamm Dis 10(3), e582 (2022). 34939346 10.1002/iid3.582 PMC8926504

[21] Dramé M, Tabue Teguo M, Proye E Should RT-PCR be considered a gold standard in the diagnosis of COVID-19? J. Med. Virol. 92(11), 2312–2313 (2020). 32383182 10.1002/jmv.25996 PMC7267274

[22] Al Bayat S, Mundodan J, Hasnain S Can the cycle threshold (Ct) value of RT-PCR test for SARS CoV2 predict infectivity among close contacts? J Infect Public Health 14(9), 1201–1205 (2021). 34416598 10.1016/j.jiph.2021.08.013 PMC8362640

[23] Tom MR, Mina MJ. To interpret the SARS-CoV-2 test, consider the cycle threshold value. J Clin Infect Dis 71(16), 2252–2254 (2020). 10.1093/cid/ciaa619 PMC7314112 32435816

[24] Rajyalakshmi B, Samavedam S, Reddy PR, Aluru N. Prognostic value of “Cycle Threshold” in confirmed COVID-19 patients. Indian J Crit Care Med 25(3), 322 (2021). 33790515 10.5005/jp-journals-10071-23765 PMC7991767

[25] Albahrani S, Alghamdi M, Zakary N Initial viral cycle threshold values in patients with COVID-19 and their clinical significance. Eur. J. Med. Res. 27(1), 101 (2022). 35765070 10.1186/s40001-022-00729-5 PMC9237989

[26] Nolan KF, Greaves DR, Waldmann HJG. The human interleukin 18 gene IL 18 maps to 11q22. 2-q22. 3, closely linked to the DRD2 gene locus and distinct from mapped IDDM loci. Genomics 51(1), 161–163 (1998). 9693051 10.1006/geno.1998.5336

[27] Giedraitis V, He B, Huang W-X, Hillert JJJON. Cloning and mutation analysis of the human IL-18 promoter: a possible role of polymorphisms in expression regulation. J. Neuroimmunol. 112(1–2), 146–152 (2001). 11108943 10.1016/s0165-5728(00)00407-0

[28] Ivanski F, Fermino BL, De Souza Cassela PLC ESTUDO DA ASSOCIAÇÃO DE POLIMORFISMOS GENÉTICOS DA IL-18 NA COVID-19. Braz J Infect Dis 26, 102593 (2022).

[29] Sun H, Guo P, Zhang L, Wang F. Serum Interleukin-6 Concentrations and the Severity of COVID-19 Pneumonia: A Retrospective Study at a Single Center in Bengbu City, Anhui Province, China, in January and February 2020. Med Sci Monit 26, e926941 (2020). 33175722 10.12659/MSM.926941 PMC7670831

[30] Zhang J, Hao Y, Ou W Serum interleukin-6 is an indicator for severity in 901 patients with SARS-CoV-2 infection: a cohort study. J Transl Med 18(1), 406 (2020). 33121497 10.1186/s12967-020-02571-x PMC7594951

[31] Giannitrapani L, Augello G, Mirarchi L Outcome predictors in SARS-CoV-2 disease (COVID-19): the prominent role of IL-6 levels and an IL-6 gene polymorphism in a western Sicilian population. J. Infect. 85(2), 174–211 (2022). 10.1016/j.jinf.2022.04.043 PMC9050196 35490738

[32] Gui-Qiang W, Lei Z, Xia W, Yan-Mei J, Fu-Sheng W. Diagnosis and treatment protocol for COVID-19 patients (tentative 8th edition): interpretation of updated key points. Infect Dis Immun 1(01), 17–19 (2021). 10.1097/ID9.0000000000000002 PMC8057309 38630067

[33] Nhc Natcm. Diagnosis and Treatment Protocol for COVID-19 Patients (Tentative 8th Edition). Infect Dis Immun 20(1), 8–16 (2020). 10.1097/01.ID9.0000733564.21786.b0 PMC8057316 38630124

[34] Ek P, Böttiger B, Dahlman D, Hansen KB, Nyman M, Nilsson AC. A combination of naso- and oropharyngeal swabs improves the diagnostic yield of respiratory viruses in adult emergency department patients. Infect Dis 51(4), 241–248 (2019). 10.1080/23744235.2018.1546055 30760088

[35] Huyut MT, Velichko A, Belyaev M. Detection of Risk Predictors of COVID-19 Mortality with Classifier Machine Learning Models Operated with Routine Laboratory Biomarkers. Appl Sci 12(23), 12180 (2022).

[36] Huyut MT, Velichko A. Diagnosis and Prognosis of COVID-19 disease using routine blood values and LogNNet neural network. Sensors 22(13), 4820 (2022). 35808317 10.3390/s22134820 PMC9269123

[37] Huyut M. Automatic Detection of Severely and Mildly Infected COVID-19 Patients with Supervised Machine Learning Models. IRBM 44(1), 100725 (2023). 35673548 10.1016/j.irbm.2022.05.006 PMC9158375

[38] Velichko A, Huyut MT, Belyaev M, Izotov Y, Korzun D. Machine Learning Sensors for Diagnosis of COVID-19 Disease Using Routine Blood Values for Internet of Things Application. Sensors 22(20), 7886 (2022). 36298235 10.3390/s22207886 PMC9610709

[39] Aksu F, Çapan N, Aksu K C-reactive protein levels are raised in stable Chronic obstructive pulmonary disease patients independent of smoking behavior and biomass exposure. J Thorac Dis 5(4), 414–421 (2013). 23991296 10.3978/j.issn.2072-1439.2013.06.27 PMC3755654

[40] Huyut MT, Ilkbahar F. The effectiveness of blood routine parameters and some biomarkers as a potential diagnostic tool in the diagnosis and prognosis of Covid-19 disease. Int Immunopharmacol 98, 107838 (2021). 34303274 10.1016/j.intimp.2021.107838 PMC8169318

[41] Xie J, Covassin N, Fan Z Association between hypoxemia and mortality in patients with COVID-19. Presented at: Mayo Clinic Proceedings. 95(6), 1138–1147 (2020). 32376101 10.1016/j.mayocp.2020.04.006 PMC7151468

[42] Yu H-H, Qin C, Chen M, Wang W, Tian D-S. D-dimer level is associated with the severity of COVID-19. Thromb. Res. 195, 219–225 (2020). 32777639 10.1016/j.thromres.2020.07.047 PMC7384402

[43] Yalçin KS, Kasapoğlu B, Alanli R, Küçükay MB, Koşar A. The association of oxygen saturation, tomography findings and D-dimer levels in coronavirus disease 2019 patients. Blood Coagul. Fibrinolysis 31(8), 558–561 (2020). 33181759 10.1097/MBC.0000000000000964

[44] Huyut MT, Huyut Z, İlkbahar F, Mertoğlu C. What is the impact and efficacy of routine immunological, biochemical and hematological biomarkers as predictors of COVID-19 mortality? Int Immunopharmacol 105, 108542 (2022). 35063753 10.1016/j.intimp.2022.108542 PMC8761578

[45] Jain A, Pandey AK, Kaur J Is there a correlation between viral load and olfactory & taste dysfunction in COVID-19 patients? Am. J. Otolaryngol. 42(3), 102911–102911 (2021). 33476975 10.1016/j.amjoto.2021.102911 PMC7836834

[46] Cho RH, To ZW, Yeung ZW COVID-19 viral load in the severity of and recovery from olfactory and gustatory dysfunction. The Laryngoscope 130(11), 2680–2685 (2020). 32794209 10.1002/lary.29056 PMC7436903

[47] Taziki Balajelini MH, Rajabi A, Mohammadi M Virus load and incidence of olfactory, gustatory, respiratory, gastrointestinal disorders in COVID-19 patients: a retrospective cohort study. Clin. Otolaryngol. 46(6), 1331–1338 (2021). 34358409 10.1111/coa.13844 PMC8444685

[48] Finsterer J, Stollberger C. Causes of hypogeusia/hyposmia in SARS-CoV2 infected patients. J. Med. Virol. 92(10), 1793–1794 (2020). 32311107 10.1002/jmv.25903 PMC7264588

[49] Buchan BW, Hoff JS, Gmehlin CG Distribution of SARS-CoV-2 PCR cycle threshold values provide practical insight into overall and target-specific sensitivity among symptomatic patients. Am. J. Clin. Pathol. 154(4), 479–485 (2020). 32687186 10.1093/ajcp/aqaa133 PMC7454307

[50] Mertoglu C, Huyut MT, Olmez H, Tosun M, Kantarci M, Coban TA. COVID-19 is more dangerous for older people and its severity is increasing: a case-control study. Med Gas Res 12(2), 51 (2022). 34677152 10.4103/2045-9912.325992 PMC8562399

[51] Griswold MG, Fullman N, Hawley C Alcohol use and burden for 195 countries and territories, 1990–2016: a systematic analysis for the Global Burden of Disease Study 2016. Lancet 392(10152), 1015–1035 (2018). 30146330 10.1016/S0140-6736(18)31310-2 PMC6148333

[52] Chakravarty D, Nair SS, Hammouda N Sex differences in SARS-CoV-2 infection rates and the potential link to prostate cancer. Commun Biol 3(1), 1–12 (2020). 32641750 10.1038/s42003-020-1088-9 PMC7343823

[53] Stack G, Jones E, Marsden M CD200 receptor restriction of myeloid cell responses antagonizes antiviral immunity and facilitates cytomegalovirus persistence within mucosal tissue. PLoS Pathog 11(2), e1004641 (2015). 25654642 10.1371/journal.ppat.1004641 PMC4412112

[54] Bwire GM. Coronavirus: Why Men are More Vulnerable to Covid-19 Than Women? SN Compr Clin Med 2(7), 874–876 (2020). 32838138 10.1007/s42399-020-00341-w PMC7271824

[55] Kishaba T, Tamaki H, Shimaoka Y, Fukuyama H, Yamashiro S. Staging of acute exacerbation in patients with idiopathic pulmonary fibrosis. Lung 192(1), 141–149 (2014). 24221341 10.1007/s00408-013-9530-0

[56] Jamal SA, Farooq MU, Bidari V. Challenges associated with using cycle threshold (Ct) value of reverse-transcription polymerase chain reaction (RT-PCR) as a criteria for infectiousness of coronavirus disease 2019 (COVID-19) patients in India. Infect. Control Hosp. Epidemiol. 43(11), 1730–1731 (2022). 34338176 10.1017/ice.2021.357 PMC8410745

[57] Antos A, Kwong ML, Balmorez T, Villanueva A, Murakami S. Unusually High Risks of COVID-19 Mortality with Age-Related Comorbidities: An Adjusted Meta-Analysis Method to Improve the Risk Assessment of Mortality Using the Comorbid Mortality Data. Infect Dis Rep 13(3), 700–711 (2021). 34449622 10.3390/idr13030065 PMC8395741

[58] Zhou F, Yu T, Du R Clinical course and risk factors for mortality of adult inpatients with COVID-19 in Wuhan, China: a retrospective cohort study. Lancet 395(10229), 1054–1062 (2020). 32171076 10.1016/S0140-6736(20)30566-3 PMC7270627

[59] Suleyman G, Fadel RA, Malette KM Clinical characteristics and morbidity associated with coronavirus disease 2019 in a series of patients in metropolitan Detroit. JAMA Netw Open 3(6), e2012270–e2012270 (2020). 32543702 10.1001/jamanetworkopen.2020.12270 PMC7298606

[60] Petrakis D, Margină D, Tsarouhas K Obesity - a risk factor for increased COVID-19 prevalence, severity and lethality. Mol. Med. Rep 22(1), 9–19 (2020). 32377709 10.3892/mmr.2020.11127 PMC7248467

[61] Faam B, Zarkesh M, Daneshpour MS, Azizi F, Hedayati M. The association between inflammatory markers and obesity-related factors in Tehranian adults: tehran lipid and glucose study. Iran J Basic Med Sci 17(8), 577–582 (2014). 25422750 PMC4240791

[62] Moriconi D, Masi S, Rebelos E Obesity prolongs the hospital stay in patients affected by COVID-19, and may impact on SARS-COV-2 shedding. Obes Res Clin Pract 14(3), 205–209 (2020). 32534848 10.1016/j.orcp.2020.05.009 PMC7269944

[63] Costela-Ruiz VJ, Illescas-Montes R, Puerta-Puerta JM, Ruiz C, Melguizo-Rodríguez L. SARS-CoV-2 infection: the role of cytokines in COVID-19 disease. Cytokine Growth Factor Rev. 54, 62–75 (2020). 32513566 10.1016/j.cytogfr.2020.06.001 PMC7265853

[64] Karra VK, Gumma PK, Chowdhury SJ IL-18 polymorphisms in hepatitis B virus related liver disease. Cytokine 73(2), 277–282 (2015). 25802197 10.1016/j.cyto.2015.02.015

[65] Pérez-Flores I, Santiago JL, Fernández-Pérez C Impacts of Interleukin-18 Polymorphisms on the Incidence of Delayed-Onset Cytomegalovirus Infection in a Cohort of Kidney Transplant Recipients. Open Forum Infect Dis 6(9), ofz325 (2019). 31660404 10.1093/ofid/ofz325 PMC6798256

[66] Sobti R, Sharma V, Abitew A IL-18 Gene promoter region 607C/A polymorphism in HIV-1 infected north indian population. Balkan J Med Genet 14(2), 41 (2011). 24052711 10.2478/v10034-011-0046-8 PMC3776698

[67] Coutinho LL, Oliveira CN, Albuquerque PL Elevated IL-18 predicts poor prognosis in critically ill COVID-19 patients at a Brazilian hospital in 2020-21. Future microbiology 17, 1287–1294 (2022). 36111789 10.2217/fmb-2022-0057 PMC9488117

[68] Chen W-J, Yang J-Y, Lin J-H Nasopharyngeal shedding of severe acute respiratory syndrome—associated coronavirus is associated with genetic polymorphisms. J Clin Infect Dis 42(11), 1561–1569 (2006). 10.1086/503843 PMC7107974 16652313

