# Supplementary figures and images for: Ct, IL-18 polymorphism, and laboratory biomarkers for predicting chemosensory dysfunctions and mortality in COVID-19
Source: Future Sci OA. 2023 Mar 9;9(2):FSO838. doi: 10.2144/fsoa-2022-0082 (PMC10005086; doi:10.2144/fsoa-2022-0082)

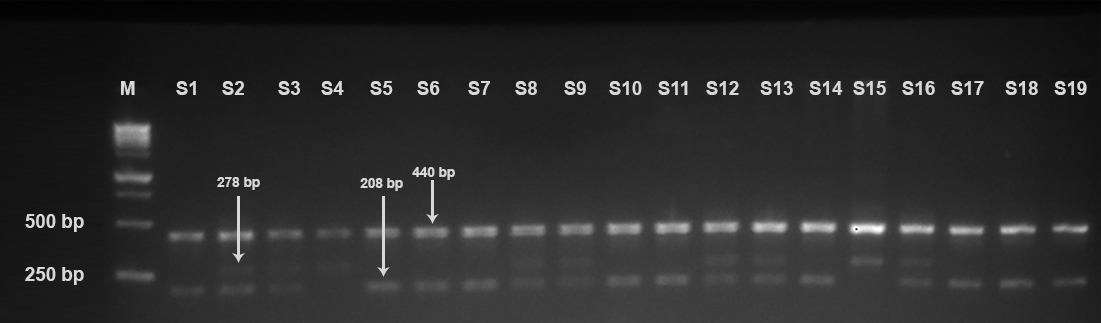

Supplement: Supplementary file 1 [file fsoa-09-838-s1.jpg]

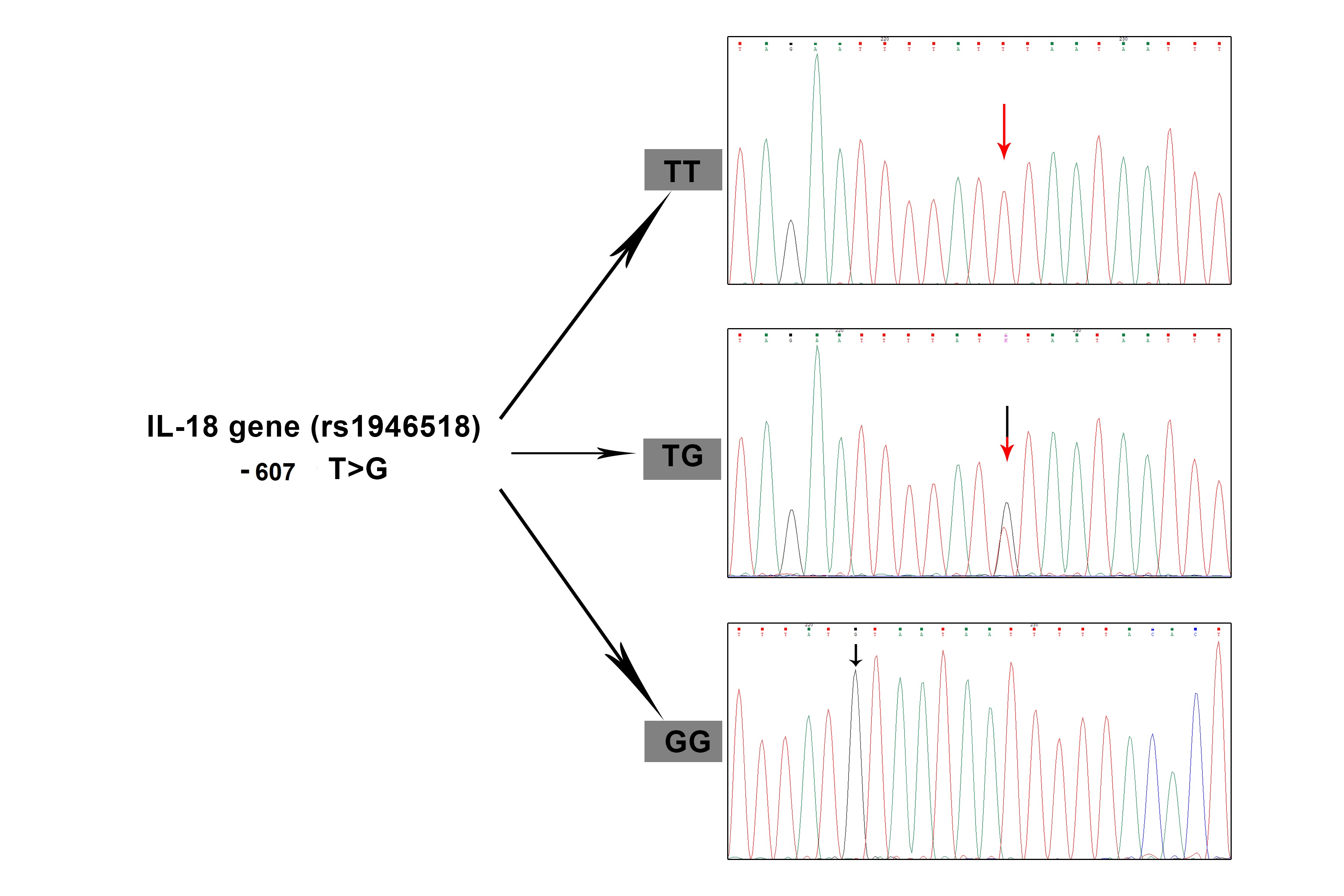

Supplement: Supplementary file 2 [file fsoa-09-838-s2.jpg]
